# Antioxidants and Cancer: Theories, Techniques, and Trials in Preventing Cancer

**DOI:** 10.1155/2018/5363064

**Published:** 2018-05-20

**Authors:** Gloria M. Calaf, Francisco Aguayo, Consolato M. Sergi, Angeles Juarranz, Debasish Roy

**Affiliations:** ^1^Instituto de Alta Investigación, Universidad de Tarapacá, Arica, Chile; ^2^Center for Radiological Research, Columbia University Medical Center, New York City, NY, USA; ^3^Departamento de Oncología Básico Clínica, Facultad de Medicina, Universidad de Chile, Santiago, Chile; ^4^Department of Laboratory Medicine and Pathology, University of Alberta, Edmonton, AB, Canada; ^5^Laboratory of Photocarcinogenesis, Department of Biology, Faculty of Sciences, Autonomous University of Madrid, Madrid, Spain; ^6^Instituto Ramón y Cajal de Investigación Sanitaria (IRYCIS), Madrid, Spain; ^7^Laboratory of Molecular Carcinogenesis, Department of Natural Science, Shirley J. Hinds Allied Health Science Complex, Hostos Community College of the City University of New York, Bronx, NY, USA

Compounds of natural origin and their derivatives play an increasingly important role in medicine and pharmacology. Approximately 60% of therapeutic drugs used in the treatment of cancer are compositions comprising natural compounds and/or their derivatives.

C. G. Vazhappilly and H. P. Vasantha Rupasinghe demonstrated that an apple flavonoid fraction (AF4) can protect oxidative DNA damage *in vitro* and facilitate repair mechanisms in normal human bronchial epithelial cells exposed to carcinogen-induced DNA damage as nicotine-derived nitrosamine ketones, nitrosamine ketones-acetate, methotrexate, and cisplatin. When DNA damage and repair mechanisms were evaluated, it was found that AF4 pretreated cells showed lower cytotoxicity, total ROS generation, and DNA fragmentation along with consequent inhibition of DNA tail moment after phosphorylation of histone (*γ*-H2AX).

A. Ortiz-Espín et al. evaluated an extract of an Antarctic plant *Deschampsia antarctica* (EDA) in young human fibroblasts exposed to H_2_O_2_ to survive in extreme conditions. They measured cell proliferation, viability, and senescence-associated *β*-galactosidase (SA-*β*-Gal). They found that EDA per se promoted cell proliferation and viability and increased the expression of antisenescence-related markers. They also tested the expression of several senescence-associated proteins including redox protein thioredoxin, sirtuin 1, and lamin A/C and the replicative protein PCNA. Then, they induced senescence in human fibroblasts and they found that an EDA treatment significantly inhibited the increase in SA-*β*-Gal levels induced by H_2_O_2_ and promoted the expression of sirtuin 1 and lamin A/C proteins. The results suggest that EDA protects human fibroblasts from cellular senescence, pointing to this compound as a potential therapeutic agent to treat or prevent skin senescence.

G. Carrasco-Torres et al. used quercetin, a flavonoid considered as chemopreventive agent in different types of cancer. They demonstrated that quercetin was able to prevent and reverse rat liver preneoplastic lesions when using the modified resistant hepatocyte model by downregulating the expression of EGFR and phosphorylating the status of Src-1, STAT5, and Sp-1. Then, they concluded that quercetin reversed preneoplastic lesions and had a chemopreventive effect on the liver of rats. Plant-based compounds are still researched for their anticancer activity and for their quantity in plants. Therefore, the modern chromatographic methods are applied to quantify them in plants, as the ultraperformance liquid chromatography-tandem mass spectrometry.

T. Kubrak et al. studied the effect of 20 coumarin derivatives on the cytotoxicity and expression of encoding proteins responsible for multidrug resistance (MDR) and genes involved in such resistance in cancer cells. Such genes are considered as the major cause of failure of cancer chemotherapy, demonstrated as overexpression of membrane transporters primarily from the ABC family which actively remove cytostatics from the tumor cell. They studied proteins as *MDR1*, *MRP*, and *LRP* and genes as *BCRP* in the presence and absence of mitoxantrone in 5 cell lines derived from the human hematopoietic system and found that leukemia cells exhibited a multidrug resistance phenotype.

M. Mehdi et al. evaluated glutamate and glucose metabolism through GDH and LDH enzyme activity, oxidant, and antioxidative status among breast cancer patients from Addis Ababa, Ethiopia. Catalytic activities of glutamate dehydrogenase, lactate dehydrogenase, and oxidative stress index were significantly increased both in serum and cancerous tissues of breast cancer patients as compared to control groups of breast cancer patients. They concluded that catalytic activities of GDH and LDH among breast cancer patients were significantly higher than control groups and noncancerous tissues of breast cancer patient. A problem of cancer chemotherapy is the high cytotoxicity toward normal rapidly proliferating cells, especially the bone marrow. In order to mitigate side effects, modified therapeutic regimens such as combination therapy have been introduced.

A. Och et al. studied the content of alkaloids as sanguinarine, berberine, protopine, and chelidonine, unidentified in plant species known for their anticancer activity. Plant-based compounds are still researched for their anticancer activity and for their quantity in plants. Therefore, the modern chromatographic methods are applied to quantify them in plants, for example, UPLC-MS/MS (ultraperformance liquid chromatography-tandem mass spectrometry).

*Gloria M. Calaf* *Francisco Aguayo* *Consolato M. Sergi* *Angeles Juarranz* *Debasish Roy*

